# Listeriosis in fattening pigs caused by poor quality silage - a case report

**DOI:** 10.1186/s12917-018-1687-6

**Published:** 2018-11-21

**Authors:** Heiko Stein, Beatrix Stessl, Rene Brunthaler, Igor Loncaric, Herbert Weissenböck, Ursula Ruczizka, Andrea Ladinig, Lukas Schwarz

**Affiliations:** 1 University Clinic for Swine, Department for Farm Animals and Veterinary Public Health, University of Veterinary Medicine, Vienna, Austria; 2 Institute of Milk Hygiene, Milk Technology and Food Science, Department of Farm Animals and Veterinary Public Health, University of Veterinary Medicine, Vienna, Austria; 3 Institute of Pathology and Forensic Veterinary Medicine, Department of Pathobiology, University of Veterinary Medicine, Vienna, Austria; 4 Institute of Microbiology, Department of Pathobiology, University of Veterinary Medicine, Vienna, Austria; 5 Current address: Vetpraxis Hegerberg, Kasten, Austria

**Keywords:** *Listeria monocytogenes*, Septicaemia, Silage, Fattening pig

## Abstract

**Background:**

*Listeria (L.) monocytogenes* as the causative agent of listeriosis in humans and different animal species, has its reservoir in the environment. It can be found in the gut and faeces of healthy pigs, but under certain circumstances it may cause clinical disease. Fatteners are usually not known to get affected by *Listeria*-associated septicaemia and enteritis. This case report shows, that *L. monocytogenes* should be part of the list of differential diagnoses, when fattening pigs suffer from haemorrhagic diarrhoea and septicaemia.

**Case presentation:**

Here, we report of an episode of fatal listeriosis in fattening pigs in a piglet producing farm in Lower Austria, which was combined with a fattening unit with space for 450 fatteners. The mortality rate resulted in 7.8% among fattening pigs after suffering from clinical symptoms such as anorexia, bloody diarrhoea and increased body temperature. Two fattening pigs with clinical symptoms and maize silage samples were used for further diagnostics. *L. monocytogenes* were isolated from serosa samples of the pigs and in the corresponding fed maize silage. One animal was positively tested for *Brachyspira hyodysenteriae*, which may have also been involved in the development of colitis. Immunohistochemically, *L. monocytogenes* could be detected in high amounts in lymphatic tissue of the gut. Molecular biological characterisation of the *L. monocytogenes* isolates from pigs and maize silage resulted in an identical DNA-fingerprint assigned to sequence type (ST) 21. Additionally, a high content of deoxynivalenol (3000 parts per billion) was found in maize silage. Therefore, the maize silage produced under inappropriate ensilaging conditions in a silo, was most likely the source of infection. Antimicrobial therapy with amoxicillin led to a fast cure of the remaining affected fatteners.

**Conclusion:**

To conclude, we were able to show, that *L. monocytogenes* can cause clinical disease in finishing pigs, which may have been a result of immunosuppression due to high deoxynivalenol exposure. When feeding silage it is important that all ensilaging procedures occur under appropriate anaerobic conditions to guarantee suppression of listerial growth.

**Electronic supplementary material:**

The online version of this article (10.1186/s12917-018-1687-6) contains supplementary material, which is available to authorized users.

## Background

*Listeria monocytogenes,* the causative agent of listeriosis in both humans and animals, is a gram-positive facultative anaerobe rod shaped bacterium with saprophytic nature [1]. *L. monocytogens* has reservoirs in agricultural landscapes, watersheds and in the farm environment [2–5]. Serotypes 1/2b, 4b (genetic lineage I) and 1/2a and 1/2c (genetic lineage II) are predominantly involved in listeriosis cases of animals and humans. Genetic recombination events occur more often in lineage II strains allowing the bacteria to successfully adapt to a broad range of environmental niches. Lineage I strains are more adapted to the human host [6]. Farm animals are reported to be carriers rather than being clinically affected by listeriosis [7]. The bovine farm ecosystem maintains a high prevalence of *L. monocytogenes*, including subtypes linked to human infections and foodborne outbreaks [8, 9]. Silage of poor quality (pH > 4.5) was reported to be an important vehicle for *L. monocytogens* transmission to cattle causing listerial encephalitis [10–12].

In peer-reviewed literature, only a few older clinical case reports have dealt with the *L. monocytogenes* transmission in pig herds despite the fact that *L. monocytogenes* contamination of pork products is of great concern for public health. Porcine listeriosis manifests mainly as septicaemia in suckling piglets, while encephalitis and abortion in sows are rarely documented [2, 13–15].

Management factors supporting the prevalence of *L. monocytogenes* in the pig farm environment are feeding wet and unheated diets or silage, a lack of hygiene barriers and change rooms for workers and weak biosecurity protocols [2, 16]. This case report provides important insights in the pathological findings and epidemiology of porcine listeriosis in fattening pigs and the most likely source of infection. Furthermore the aim of this case report was to highlight the importance of *L. monocytogenes* in diagnostics of enteritis in fattening pigs.

## Case presentation

### Anamnesis

**Fig. 1:**
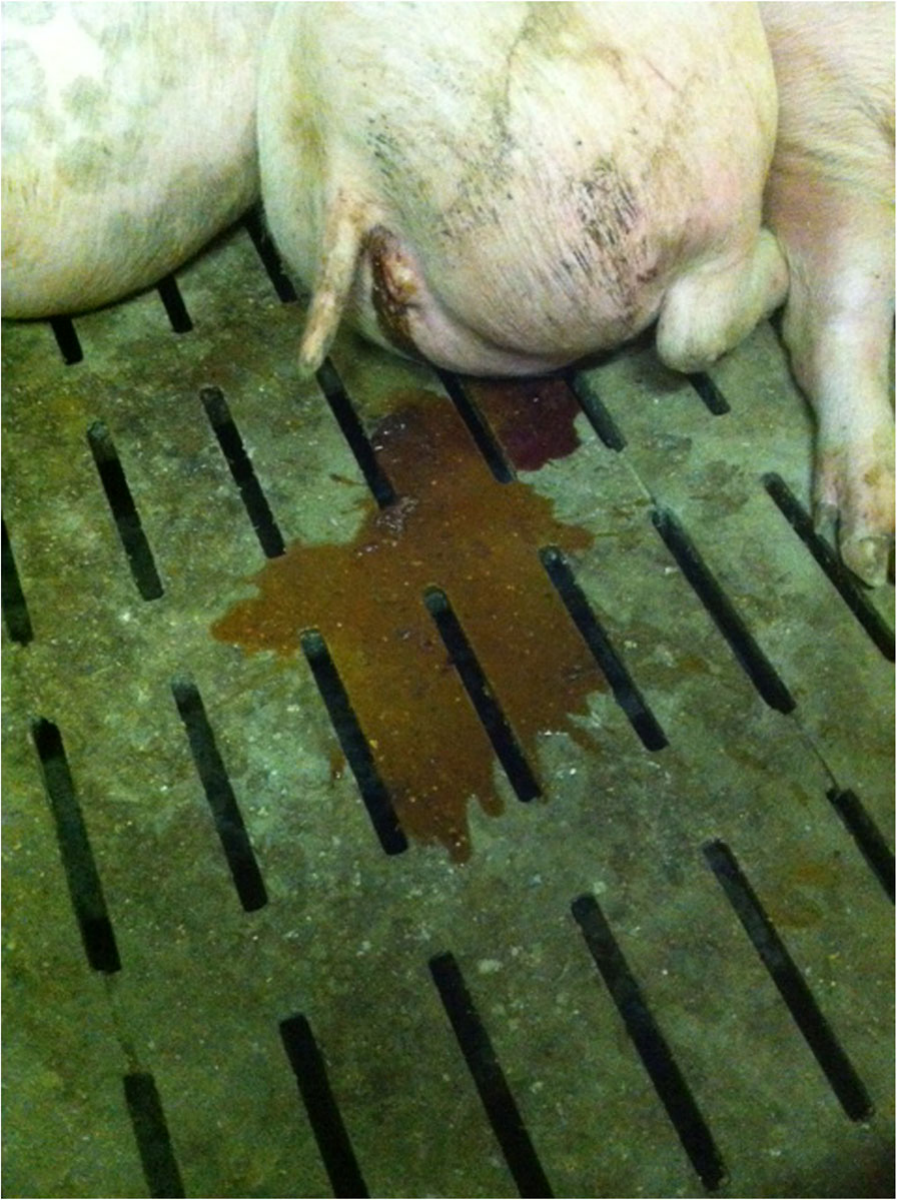
One of the affected fatteners loosing bloody and watery diarrhoea while resting

The case herd was located in Lower Austria and was producing piglets with 60 sows combined with an integrated fattening unit with space for 450 pigs. Fattening pigs were produced continuously and were kept in one building subdivided in six barns each with about 75 fattening pigs. Anorexia, bloody diarrhoea (Fig. 1), and an increased body temperature up to 40 °C (range from 39.5–40 °C) were observed by the responsible farm veterinarian in about 10% of fattening pigs, mostly in well-fed animals of 40–100 kg of bodyweight. The number of affected animals was estimated by the herd veterinarian retrospectively. In total, 35 fattening pigs (7.8%; normal mortality rate in that weight classes < 2%) of one batch died in a time period of approximately 3 weeks. Animals were fed a maize silage based diet via a spotmix multiphase feeding system. Additionally, the farmer reported a change of the batch of maize silage used for feeding the finisher pigs. To evaluate the involvement of mycotoxins in this case, the responsible farm veterinarian took one sample of the remaining maize silage, which was fed at the moment when pigs died acutely and one sample of the new batch. Both samples were sent to an external laboratory for analysis (ELISA) of the two major and commonly found mycotoxins zearalenon (ZEA) and deoxynivalenol (DON). The maize silage was contaminated with 3000 parts per billion (ppb) DON whereas ZEA was detected at a level of 270 ppb. Both values are not considered harmful. Previously to our investigations, necropsy at the animal cadaver utilization of dead fatteners resulted in haemorrhagic colitis most probably linked to swine dysentery. Since no molecular biological investigations were initiated by the herd veterinarian to find out the causative agent of the dead animals, he forwarded two characteristically sick and living pigs (pig 1: female, 39.5 kg bodyweight; pig 2: female, 105 kg bodyweight) and the maize silage samples to the University Clinic for Swine, University of Veterinary Medicine Vienna, for further diagnostics approximately three weeks after the first dead fattening pigs were observed.

### Clinical examination

On the next day pig 1 was still alive whereas pig 2 had died over night. Pig 1 was clinically examined and showed apathy, a dog-sitting position and subsequently during the examination the pig was lying in lateral recumbency. The conjunctivae were moderately reddened, the rectal body temperature was 38.7 °C, during the auscultation of the heart a continuous heart murmur was determined; the heart rate was 140 beats per minute. The ears, the snouts and the skin of the hind limbs of both animals were cyanotic. The clinical findings led to a suspected diagnosis “septicaemia” and/or “circulatory insufficiency”. After the clinical examination, the pig was first anesthetized by an intravenous administration of ketamine (10 mg/kg body weight (BW), Narketan®, Vetoquinol AustriaGmbH, Vienna, Austria) and azaperon (2.5 mg/kg BW, Stresnil®, Elanco Animal Health, Bad Homburg, Germany) and finally euthanized by an intracardial administration of a combination preparation of embutramide, mebezonium iodide and tetracaine hydrochloride (1 ml/10 kg BW, T61®, Intervet GmbH, Vienna, Austria).

### Necropsy, pathohistological examination and immunohistochemistry

Both pigs were necropsied and tissue samples (heart, lung, liver, spleen, kidney, one piece each of the jejunum, ileum and ascending colon, pancreas and inguinal lymph nodes) were routinely processed for histopathological and immunohistochemical investigations. Tissue samples were fixed in 10% formalin, alcohol dehydrated and embedded in paraffin wax. Sections were cut and stained with haematoxylin-eosin and examined by light microscopy.

**Fig. 2:**
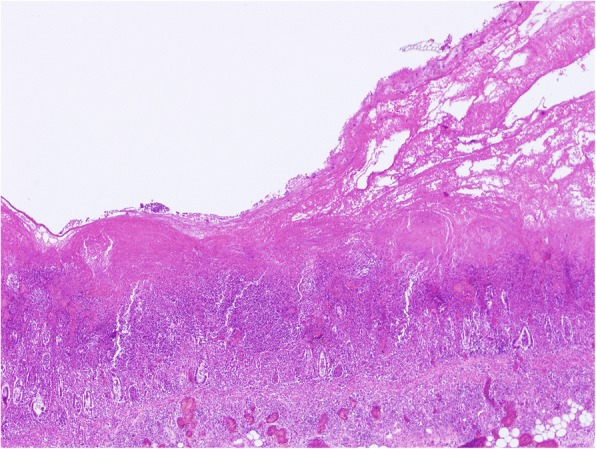
Severe fibrinonecrotic colitis, pig 1 (Hematoxylin and Eosin staining)

Macroscopically except for a high-grade alveolar pulmonary oedema no appreciable pathological alterations were detected in the thoracic organs. The stomach was well filled with inconspicuous feed. In both animals the most striking finding was a severe diffuse fibrinonecrotic typhlocolitis (Fig. 2) with large amounts of intralesional bacteria. Pig 2 additionally showed a moderate diffuse fibrinous serositis especially in the area of the intestinal tract. The rest of the abdominal organs showed no pathological changes (Table 1). The mucous membranes of the stomach and small intestine were macroscopically and histologically unremarkable. As *L. monocytogenes* was not a possible differential diagnosis and clinical symptoms did not let us assume an affection of the central nervous system, the brain, meninges and spinal cord were not investigated pathologically.

**Table 1:** Summary of the main findings

		Pig 1	Pig 2	Silage
Pathoanatomical and pathohistological findings	thorax	low grade dystelectasis, low grade peribronchial interstitial inflammatory round cell infiltration	high-grade pulmonary oedema, low grade peribronchial interstitial inflammatory round cell infiltration	n.a.
	abdomen	severe diffuse fibrinonecrotic typhlocolitis, remaining abdominal organs (including inguinal lymphnodes) without pathological findings	severe diffuse fibrinonecrotic typhlocolitis; moderate diffuse fibrinous serositis in the area of the intestine	n.a.
	IHC *L. monocytogenes*	positive in: lymphatic tissue, lamina muscularis mucosae and lamina propria mucosae of the large intestine; spleen, inguinal lymph nodes		n.a.
Triplex-PCR		positive (*B. hyodysenteriae*)	negative	n.a.
Microbiological examination of serosa		*L. monocytogenes* (Pig 1 + 2; Silage)		
PFGE analysis of *L. monocytogenes*		PCR-serogroup 1/2a, 3a (Pig 1 + 2; Silage)		
MLST of *L. monocytogenes* isolates		ST21 (Pig 1 + 2; Silage)		
AST of *L. monocytogenes* isolates	AMP (0.12 to 16 μg/ml)^a^	sensitive (≤0.12)^b^	sensitive (≤0.12)^b^	sensitive (≤0.12)^b^
	PEN (0.06 to 8 μg/ml)^a^	sensitive (0.25)^b^	sensitive (0.12)^b^	sensitive (0.1)^b^
	SXT (0.5/9.5 to 4/76 μg/ml)^a^	sensitive(≤0.5/≤9.5)^b^	sensitive(≤0.5/≤9.5)^b^	sensitive(≤0.5/≤9.5)^b^

^a^Values in brackets indicate the test range of according antimicrobial substance

^b^Values in brackets represent tested concentration of antimicrobial substance. *AST* = antimicrobial susceptibility testing, *n.a*. = not applicable

**Fig. 3:**
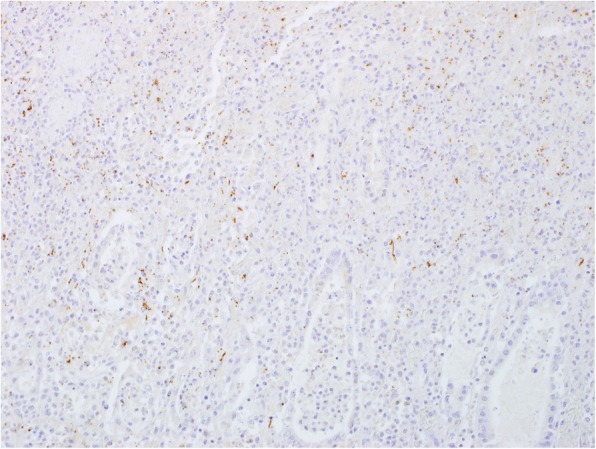
Distribution of *Listeria monocytogenes* antigen (brown signals) in the lamina propria mucosae of the colon, pig 1 (*L. monocytogenes* immunohistochemistry)

For demonstration of listeria, sections of ascending colon with evident and characteristic histopathological lesions were subjected to immunohistochemistry (IHC) using a rabbit polyclonal antiserum raised against whole cell antigens of *L. monocytogenes* (Difco, Sparks, MD, USA). Immunohistochemical investigations were performed using the HRP polymer method on a Lab Vision-Autostainer (Thermo Fisher Scientific, Fremont, CA, USA). Formalin fixed, paraffin embedded tissue samples were sectioned (2 μm) and placed on positively charged glass slides (Superfrost plus; Menzel Glaeser, Braunschweig, Germany). They were dewaxed in Neo Clear® solution (Merck, Darmstadt, Germany) and rehydrated two times in a series of alcohols (100, 96, and 70%) and distilled water. After dewaxing and rehydration, antigen retrieval was done by heating the slides in citrate buffer (pH 6.0) in the Lab Vision PT Module (Thermo Fisher Scientific). The slides were then incubated in Hydrogen Peroxidase Block (Thermo Fisher Scientific) for 5 min, followed by 10-min incubation in Ultravision Protein Block (Thermo Fisher Scientific), to diminish nonspecific background staining. Afterwards sections were incubated with the primary antibody (dilution 1:6000) for 30 min at room temperature, followed by the Primary Antibody Enhancer (Thermo Fisher Scientific) for 15 min, and finally by Ultra Vision Large Volume HRP Polymer (Thermo Fisher Scientific) for 20 min. The signal was visualized with Diaminobenzidine (DAB) Quanto Substrate System (Thermo Fisher Scientific) for 5 min. After the staining reaction, the slides were counterstained with 1:20 diluted Mayer ´s Hematoxylin (Thermo Fisher Scientific) for 1 min, dehydrated in a series of graded alcohols (70, 96, and 100%) and treated with Neo Clear® (Merck, Darmstadt, Germany). Finally, the slides were mounted with Neomount (Merck, Darmstadt, Germany) for microscopic examination. This examination revealed large amounts of intracellular bacterial antigen in the large intestine, where it was localized within diffusely distributed lymphatic tissue, in the lamina propria mucosae and in scattered foci of the lamina muscularis mucosae (Fig. 3, Table 1). Furthermore, some positive signals were present in scattered cells of the splenic pulp. A positive reaction was also found in the inguinal lymph node.

In pig 1 a triplex polymerase chain reaction (PCR) of a mucosal scraping of the ileum and the colon for simultaneous detection of *Lawsonia intracellularis*, *Brachyspira (B.*) *hyodysenteriae* and *B. pilosicoli* (according to La et al., 2006) gave a positive result for *B. hyodysenteriae* [17], whereas pig 2 was negative for all three pathogens (Table 1).

### Microbiological examination and molecular typing of isolated *Listeria monocytogenes*

A part of peritoneal serosa of both pigs was submitted to bacteriological and mycological examination. After cultivation of 24 h at 37 °C on three different routinely used media [blood agar (BA) BD Columbia Blood Agar with 5% *v*/v sheep blood in aerobic/microaerophilic (5% CO2)/anaerobic conditions, and Columbia CNA Improved II agar (CNA) with 5% v/v sheep blood and BD MacConkey II (all from Becton Dickinson, Germany)] small weakly beta-hemolytic colonies were observed on BA and CNA. Species identification was performed by matrix-assisted laser desorption-ionization–time of flight mass spectrometry (MALDI-TOF MS) (Bruker Daltonik, Bremen, Germany) resulting in *L*. *monocytogenes*. Silage samples were investigated according to the ISO 11290-1 protocol [18]. A sample size of 25 g or ml was homogenized and enriched in 225 ml Half-Fraser broth for 24 h at 30 °C (HFB; Merck KgA, Darmstadt, Germany). HFB (0.1 ml) was transferred to 10 ml Fraser broth (FB; Merck KgA) and incubated for 48 h at 37 °C. Primary and secondary enrichment (HFB and FB) were streaked on Agar *Listeria* acc. to Ottaviani and Agosti (ALOA; Merck KgA, Darmstadt, Germany) and Palcam agar (Biokar Diagnostics, Beauvais Cedex, France). Suspicious *L. monocytogenes* colonies were isolated from selective Listeria agar plates (ALOA and Palcam) and subcultivated on Tryptone soy agar supplemented with 0.6% (*w*/*v*) yeast extract (TSAY; Oxoid Ltd., Hampshire, UK). *L. monocytogenes* isolates were cryo-conserved in 15% glycerol stocks (Merck KgA, Darmstadt, Germany) at − 80 °C in the *Listeria* collection of the Institute of Milk Hygiene, Milk Technology and Food Science (Vetmeduni, Vienna, Austria). *L. monocytogenes* PCR -serotyping was conducted after DNA extraction including Chelex® 100-Resin (BioRad, Hercules, CA, USA), as described by Walsh et al. (1991) [19]. The *Listeria*-specific *prs* is present in all *Listeria* species. *L. monocytogenes* PCR-serogroup 1 (serovar 1/2a, 3a) is characterized by the amplification of lmo0737; PCR-serogroup 2 (serovar 1/2c, 3c) contains lmo0737 and lmo1118; In PCR-serogroup 3 (serovar 1/2b, 3b, 7) ORF2819 is present and in PCR-serogroup 4 both ORF2819 and ORF2110 Doumith et al. [20] According to Leclercq et al. [21] PCR-serogroup 5 (subgroup of 4 b isolates) is characterized by the presence of ORF2819, ORF2110 and lmo0737.

The PFGE analysis of *L. monocytogenes* was performed according to the PulseNet protocol (http://www.pulsenetinternational.org/protocols/pfge/; [22–24]) applying restriction enzyme *Asc*I and *Apa*I (each 50 U/per plug) for 4 h at 37 °C and 25 °C. Pulsed field electrophoresis run conditions were as follows: plugs were inserted in a 1% SeaKem gold agarose gel and separated in 0.5× Tris-borate EDTA (TBE) buffer at 6 V/cm with pulse times from 4.0 to 40.0 s at 14 °C and an included angle of 120° for 22.5 h (Chef DR III system; Bio-Rad Laboratories, Inc., Hercules, CA, USA).

PFGE gels were stained with ethidium bromide (Sigma-Aldrich), photographically documented as TIFF images and analyzed with the BioNumerics 6.6 software (Applied Maths NV, Sint-Martens-Latem, Belgium).In the PFGE cluster analysis the unweighted pair group mathematical average (UPGMA) algorithm including a Dice similarity coefficient and position tolerance of 1.5% was applied.

Multilocus sequence typing (MLST) followed the protocol as previously described [25]. Allelic profiles representing seven *L .monocytogenes* specific housekeeping genes were assigned by comparing the consensus sequences with the information already published in the Institute Pasteur *L. monocytogenes* MLST database [26]. Clonal complexes (CC) are groups of STs with only one housekeeping gene as major difference [25].

**Fig. 4:**
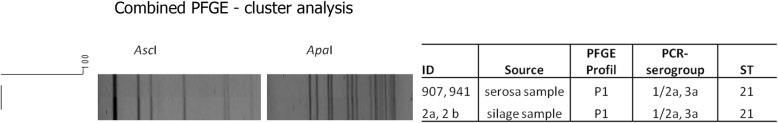
Combined PFGE-cluster analysis of *L. monocytogenes* isolated from serosa and silage samples

*L. monocytogenes* isolated from silage and serosa were confirmed as PCR-serogroup 1/2a, 3a and indicated an identical PFGE-profile in the combined cluster analysis with *Asc*I and *Apa*I (Fig. 4). Furthermore, MLST analysis revealed that the *L. monocytogenes* isolates were assigned to sequence type (ST) 21 (clonal complex CC21) (Table 1, Additional file 1).

The antimicrobial susceptibility of *L. monocytogenes* isolated from silage and pleura liquid was tested according to CLSI document M45 [27]: ampicillin (AMP; 0.12 to 16 μg/ml), penicillin G (PEN; 0.06 to 8 μg/ml) and trimethoprim-sulfamethoxazole (SXT; 0.5/9.5 to 4/76 μg/ml). Antimicrobial susceptibility testing of the *L. monocytogenes* ST21 isolated from serosa and silage showed susceptibility to all tested antimicrobials (Table 1).

### Silo inspection

After the results indicated a *L. monocytogenes* contamination of maize silage and clinical cases among fattening pigs, a farm visit was performed to further inspect the situation directly on farm. Especially the silos were inspected regarding their condition. In total four silos were used for maize silage production. Three were made of concrete and one was made of fibre-reinforced plastic. Each cemented silo was accessible via metal doors, which were tightened with plastic seals. Plastic seals were renewed every year. The one fibre-reinforced plastic silo had integrated doors, which were provided with rubber seals. These seals had not been renewed since the silo was built and had been used for several years. Rubber seals of the fibre-reinforced plastic silo showed moderate to severe signs of wastage. Since the doors to access the silo had not been additionally sealed with plastic, a continuous source of fresh air may have resulted in insufficient ensiling of maize at least in the area of the doors. This could be confirmed by the farmer, who reported a severe mould contamination of maize silage around the area of the doors.

### Therapeutic and prophylactic measures

After confirmation of the diagnosis listeriosis two days after the animals were brought to us, two more fatteners died and the responsible herd veterinarian was advised to treat all fattening pigs with amoxicillin orally via feed (20 mg/kg bodyweight for 5 days, Amoxi-Mix 100 mg/g Pulver, AniMed Service AG, Dobl, Austria). Amoxicillin was chosen, because it was assumed that due to susceptibility testing β-lactam antibiotics (such as ampicillin) generally should be efficacious. Moreover amoxicillin could be administered as in-feed medication. After the start of the antibiotic treatment, no further deaths occurred and haemorrhagic diarrhoea disappeared within two days. Furthermore, the farmer was informed to discard the remaining maize silage of the one fibre-reinforced plastic silo to avoid reinfections with *L. monocytogenes*. Prophylactically, adequate cleaning and disinfection of animal units and of all silos after they were emptied was implemented using disinfectants based on peracetic acid. Due to the zoonotic potential of *L. monocytogenes*, we recommended to follow strict personal hygienic measures such as cleaning and disinfecting hands after contact with pigs, faeces and maize silage and changing of working clothes followed by showering.

## Discussion and conclusion

The present case is the first report on clinical listeriosis in fattening pigs with some details on pathology and epidemiology. Listeriosis in pigs is a rarely diagnosed disease and usually is not considered by veterinarians [28]. The septicaemic form of listeriosis in piglets is the most commonly described form [14, 29, 30]. Diarrhoea and haemorrhages in the small intestine were seen in two piglets in a case report of Hale [31]. Before suckling piglets died, the lactating sows showed anorexia, reduced lactation and lethargy [32]. Also, anorexia could be observed in an experimental oral infection of piglets with *L. monocytogenes* [33]. *L. monocytogenes* could be isolated from liver, spleen, mesenteric lymph nodes, kidney, heart and heart blood [13, 29, 31, 32, 34]. A bacteraemia was observed 12–36 h after oral infection [33]. Focal necrosis of the liver, hepatomegaly and inflammatory infiltrates of lymphocytes and macrophages have been described as characteristic lesions [13, 14, 29, 31–34].

Moderately enlarged lymph nodes were reported in two cases [14, 32], which could not be confirmed in our case. But immunohistochemical staining showed a positive reaction for *L. monocytogenes* in the inguinal lymph node. The main pathologic lesions were focal necrosis of the Peyer’s patches, ulcers in colon, necrosis of the epithelium, which affected just the villous tips [33]. In our case we observed a necrotizing typhlocolitis in both pigs but only in pig 1 we detected *Brachyspira hyodysenteriae*, which may be one explanation of the anamnestically reported bloody diarrhoea and pathoanatomically observed necrotizing typhlocolitis. Nevertheless, since the role of *B. hyodysenteriae* in this case is not clear, we rather hypothesize, that *B. hyodysenteriae* in pig 1 just may have indicated a co-morbidity event, because no efficacious anti-brachyspiral disinfectant was used for sanitation. One would expect ongoing dysentery events in the herd without implementation of *Brachyspira*-specific control measures.

Immunohistochemical staining showed positive signals for *L. monocytogenes* in the spleen. Further lesions such as lung oedema and interstitial pneumonia with macrophage infiltration in alveolar septa were described [13, 14, 33]. A lung oedema was observed in pig 2 and interstitial round cell infiltrates were seen in the lungs of both pigs. Pig 1 also had a slight dystelectasis of the lung. Hale, Lopez and Bildfell observed similar lesions and also observed lung congestion [14, 31]. Pleural and pericardial effusion, fibrinous inflammation in peritoneum were seen in several case reports in piglets [13, 14, 32], as well as in pig 2.

In an experimental *Listeria* infection in piglets ulcers were observed especially in the colon [33]. The detection of *L. monocytogenes* in all parts of the intestine by immunohistochemistry supports the hypothesis that *L. monocytogenes* may play a crucial role in haemorrhagic diarrhoea even though there is no information regarding pathophysiological mechanisms. In horses *L. monocytogenes* can cause a necrotizing typhlocolitis [35]. Clark et al. (2004) described gastroenteritis in sheep associated with *L. monocytogenes,* similar to enteric salmonellosis [36]. Hence, necrotizing typhlocolitis in both pigs of this case may have been caused by *L. monocytogenes* itself.

The source of *L. monocytogenes* infection was maize silage additionally contaminated with 3000 ppb DON and 270 ppb ZEA produced by *Fusarium* spp.. Chronic exposure to mycotoxins, even in harmless concentrations, in silage leads to unspecific immune-system suppressions, an increased risk for infections and metabolic and hormonal dysfunction [37, 38]. In combination with the *L. monocytogenes* contaminated maize silage the fattening pigs may have developed an enteric listeriosis, which resulted in necrotizing typhlocolitis and consequently in a septicaemic listeriosis. It has been described that DON impairs the gut barrier function and increases the permeability of the gut. The main cytotoxic effect of DON in the intestine is the modulation of the local mucosal immune response, which leads to an immunosuppression or stimulation of local immune cells and cytokine release and therefore supports persistence and pathogenicity of enteropathogens in the intestine [39].

The risk for *L. monocytogenes* contamination in silage is increased in mold contaminated batches with pH values > 4.5 [10, 40]. Weis (1973) reported cases of septicaemia in sows after the intake of silage, but these cases are not well documented [41]. In outbreaks of enteric listeriosis in steers and in a case of nervous listeriosis in a bull, spoiled silage and the pasture were found as the sources of infection, respectively [42, 43]. In sheep with septicaemia and nervous form of listeriosis, soil and water of the environment were identified as origin of infection [44]. Good hygiene and manufacturing practices during silage processing are mandatory.

Food business operators are responsible for the safety of products from primary production (pre-harvest level) to secondary production (harvest level) (EC regulation 178/2002 and 852/2004) [45, 46]. The risk of *L. monocytogenes* spread in the primary production should be estimated applying culture-dependent (ISO 11290) and culture-independent methods (e.g. real-time PCR) to prevent the dissemination to farm animals, secondary food processing and in the worst case to the consumers [18, 47–49]. Research is needed to develop novel and fast diagnostic techniques to help veterinarians recognize the relationship between mycotoxin producing molds in contaminated feeding stuffs and the high risk for listeriosis and other systemic infections caused by pathogenic bacteria [50]. Acidification of silage with organic acids to reduce the pH-value in order to inhibit the growth of listeria should be a standard measure in processing silage [51, 52].

In animals and humans, the most common *L. monocytogenes* infections are caused by three serotypes: 1/2a, 1/2b, and 4b. PCR-based serotyping of the *L. monocytogenes* isolates from pig serosa and silage of this case resulted in serotype 1/2a. Genetic lineage II strains (serotype 1/2a and 1/2c) are highly abundant in the meat processing chain and seem to be better adapted to stress factors due to the presence of transposons, prophages and plasmids [53, 54]. The molecular epidemiological analysis revealed that *L. monocytogenes* isolates from the animals and isolates from silage, were clonal in the PFGE results and could be assigned to ST21. ST21 is globally not widespread and primarily originated from wild animals e.g. fox, hare and wild birds [26]. Therefore, beside specific farm management factors (e.g. small group sizes, manure treatment, coarse feed, and hygienic drinking trough design) an efficient disease management is important [2].

Boscher et al. (2012) identified similar serotypes and genotypes in clinically unaffected sows and fattening pigs suggesting common sources of contamination [55]. This is in agreement with our study, where silage was identified as the most plausible source of *L. monocytogenes* transmission. Especially farms with weak hygiene management and biosecurity measurements lead to increased *L. monocytogenes* prevalence [56]. In this case deficits regarding the silage processing seemed to be of crucial importance. As just the fibre-reinforced plastic tank was not additionally sealed with plastic, the continuous source of fresh air around the entrance doors may have caused higher pH values due to insufficient ensilaging and therefore may have enhanced moulding and growth of *L. monocytogenes*.

Healthy fatteners often carry *L. monocytogenes* and the tonsils seemed to be a high-risk tissue for cross-contamination of pork meat during slaughtering [57]. SARNO et al. (2015) investigated tonsil samples at the abattoirs and found among a large variety of genotypes also isolates assigned to ST21, the predominant ST in our case [58].

The results for antibiotic susceptibility testing revealed that examined strains were non-resistant and treatment with e.g. beta-lactam antimicrobials should have been effective [59]. Nevertheless, since the antimicrobial therapy using amoxicillin was successful and beta-lactam antimicrobials are not routinely used for treatment of brachyspiral infections, we hypothesize that necrotizing typhlocolitis was initially caused by *L. monocytogenes* and secondary aggravated by *B. hyodysenteriae*.

To conclude, we were able to show, that *L. monocytogenes* is able to cause clinical disease in fattening pigs, which may have been linked to mycotoxin-caused immunosuppression. Whenever bloody diarrhoea combined with increased mortality occurs in fattening pigs, *L. monocytogenes* should be part of the list of differential diagnoses, especially if silage is part of feeding. Since in our case therapeutic and prophylactic measures led to a fast recovery of diseased animals, antimicrobial therapy is indicated as soon as possible to avoid further shedding of *L. monocytogenes* via faeces and ongoing fatal cases in the remaining population.

## Additional file

Additional file 1: Multi-locus sequence typing – raw sequence data (Fasta format). (DOCX 14 kb)

## Abbreviations

(*w*/*v*): Weight per volume
°C: Degree of Celsius
AMP: ampicillin
AST: Antimicrobial susceptibility testing
BA: Blood agar
bp: Base pairs
BW: Body weight
CC: Clonal complex
CNA: Columbia CNA Improved II agar
DNA: Deoxyribonucleic acid
DON: Deoxynivalenol
FB: Fraser broth
g: Gram
h: Hour
HFB: Half-Fraser broth
HRP: Horseradish peroxidase
IHC: Immunohistochemistry
kg: Kilogram
M: Mol
MALDI-TOF MS: Matrix-assisted laser desorption-ionization–time of flight mass spectrometry
mg/g: Milligram/gram
mg/kg: Milligram/kilogram
min: Minutes
ml: Milliliter
MLST: Multi-locus sequence typing
PCR: Polymerase chain reaction
PEN: Penicillin
PFGE: Pulse field gel electrophoresis
ppb: Parts per billion
ST: Sequence type
SXT: Sulfamethoxazole-trimethoprim
TBE: Tris-borate EDTA
TIFF: Tag image file format
U: Unit
UPGMA: Unweighted pair group mathematical average
V/cm: Volt/centimeter
ZEA: Zearalenon
μg/ml: Microgram/milliliter
μL: Microliter
μM: Micro Mol
μm: Micrometer
