# Supplementary material for: Listeriosis in fattening pigs caused by poor quality silage - a case report
Source: BMC Vet Res. 2018 Nov 21;14:362. doi: 10.1186/s12917-018-1687-6 (PMC6249783; doi:10.1186/s12917-018-1687-6)
Supplement: Supplementary file 1 — Multi-locus sequence typing – raw sequence data (Fasta format). (DOCX 14 kb) [file 12917_2018_1687_MOESM1_ESM.docx]

|  | **ID** | **abcZ** | **bglA** | **cat** | **dapE** | **dat** | **ldh** | **lhkA** | **ST** |
| --- | --- | --- | --- | --- | --- | --- | --- | --- | --- |
| Silage sample | SW2a | 7 | 7 | 3 | 10 | 5 | 6 | 1 | 21 |
| Serosa sample | SW941 | 7 | 7 | 3 | 10 | 5 | 6 | 1 | 21 |

>SW2a_abcZ7

TTATGACAGGGGTAGCTGGTAAATCGACGAACAGAATGCGTATAGGGCTTTTCCGCAAGATGGAAAAACTATCGATTCGTTTCTTCGATAGCCGCAATGATGGCGAAATGCTTAGCCGCTTCACTAGTGACTTAGATAATATTTCCAATACACTAAACCAAGCATTGATCCAAGTACTATCCAACGTCGCGCTAATGATTGGTGTTATCATCATGATGTTCCAACAAAACGTGGAACTAGCCTTCGTTACTCTAATATCTGCTCCATTTGCAATTATTATTGCGACAGTGATTATTCGAAAAGCACGTAAATTCGTTGATGTTCAACAAGATGAACTAGGCGTACTTAACGGCTACATTGACGAAAAAATCTCTGGACAAAAAATCATTATCACAAATGGTTTAGAAGAAGAAACAATTGACGGCTTTGTTAAACAAAACAATATCGTTAAAAACGCCACTTATAAAGGGCAAGTTTACTCCGGTTTACTTTTCCCAATGATGCAAGGTATTTCCCTATTAAATACAGCTATCGTTATCTTCTTCGGTGGATGGTTAGCTCTAAACGGCGACCTTGAGAAATTGT

>SW2a_bglA7

GCTGCTGCAACCAATTCGAAGGCGCTTACAACGTCGATGGAAAAGGACTTTCCGTTCAAGATGTTACTCCAAAAGGCGGATTCGGTCACATTACTGACGGTCCAACACCAGATAACTTAAAATTAGAAGGAATCGACTTCTATCACCGTTACAAAGATGACGTGAAACTTTTTGCTGAAATGGGCTTCAAAGTTTTCCGTACTTCCATCGCTTGGTCCCGTATCTTCCCAAATGGTGACGAAACAGAGCCAAACGAAGCAGGACTAAAATTTTACGATGATTTATTCGACGAACTTCTAGCACATAATATCGAACCACTGATTACTTTATCTCATTATGAAACACCACTTCACTTATCGAAAGCTTACGACGGATGGGTAAATAGAAAAATGATCGACTTCTATGAAAACTATGTCCGCACCGTATTTAATCGGAAATTGT

>SW2a_cat3

GGGTTGTACATGCTCGTGGTGCTGGTGCGCACGGGAAATTTGTTACTAAAAAAAGCATGAAAAAATATACAATGGCTAAATTTTTGCAAGAAGAAGGGACGGAAACAGAGGTTTTTGCTCGTTTTTCAACTGTAATTCATGGACAACATTCTCCAGAAACATTACGTGATCCACGAGGTTTCTCCGTTAAGTTTTATACGGAAGAGGGAAATTATGACTTTGTCGGAAATAATTTGCCAGTATTTTTCATTCGTGATGCGATTAAGTTTCCAGATGTTATTCATTCCTTGAAGCCTGACCCGCGCACAAATATTCAAGATGGCAATCGTTACTGGGATTTCTTTAGCCTTACACCGGAAGCTACGACGATGATTATGTACTTATTCAGTGATGAAGGAACGCCGGCTTCTTACCGCGAAATCCGGGGCTCTAGTGTTCATGCGTTCAAATGGATTAACGAAGAAGGCAAAACAGTTTATGTAAAATTGCGCTGGGTTCCAAAAGCAGGAATCGTCAATCTGAAATTG

>SW2a_dapE10

TTGCGAACTATTTGCAAAGTTGTTAGCTGAACATGGTATTGAGTCCGAAAAGGTACAATACGACGTAGACAGAGCTAGCCTAGTAAGCGAAATTGGTTCCAGTAACGAGAAGGTTTTGGCATTTTCAGGGCATATGGATGTAGTTGATGCGGGTGATGTATCTAAGTGGAAGTTCCCACCTTTTGAAGCGACAGAGCATGAAGGGAAACTATACGGACGCGGCGCAACGGATATGAAGTCAGGTCTAGCGGCGATGGTTATTGCAATGATTGAACTTCATGAAGAAAAACAAAAACTAAACGGCAAGATCAGATTATTAGCAACAGTTGGGGAAGAAATCGGTGAACTTGGAGCAGAACAACTAACACAAAAAGGTTACGCAGATGATTTAGATGGTTTAATCATCGGCGAACCGAGCGGACACAGAATCGTTTATGCGCATAAAGGTTCCATTAATTATACCGTTAAATCCACTGGTAAAAATGCCCATAGTTCGATGAAATTGT

>SW2a_dat5

CCGCGGATATCAGTTTGGTGATGGTGTATATGAAGTAGTTCGTCTATATAATGGAAAATTCTTTACTTATAATGAACACATTGATCGCTTATATGCTAGTGCAGCAAAAATTGACTTAGTTATTCCTTATTCCAAAGAAGAGCTACGTGAATTACTTGAAAAATTAGTTGCCGAAAATAATATCAATACAGGGAATGTCTATTTACAAGTGACTCGTGGTGTTCAAAACCCACGTAATCATGTAATCCCTGATGATTTCCCTCTAGAAGGCGTTTTAACAGCAGCAGCTCGTGAAGTACCTAGAAACGAGCGTCAATTCGTTGAAGGTGGAACGGCTATTACAGAAGAAGATGTGCGCTGGTTACGCTGTGATATTAAGAGCTTAAACCTTTTAGGAAATATTCTAGCAAAAAATAAAGCACATCAACAAAATGCTTTGGAAGCTATTTTACATCGCGGGGAACAAGTAACGGAATGTTCTGCTTCAAACGTTTCTATTATTAAAGATGGTGTATTATGGACGCAGAAATTGT

>SW2a_ldh6

GGGGATGCAATGGATTTAAGCCATGCCGTTCCATTTTCTACACCGAAGAAAATCTACTCAGCAAATTATAGCGACTGCCACGATGCGGACCTAGTTGTTGTAACTGCCGGTACTGCTCAAAAACCTGGTGAAACTCGTTTAGATCTAGTAAATCGTAATATCAAAATCATGAAAGGCATCGTGGATGAAGTTATGGCAAGCGGATTTGATGGCATCTTCTTAATCGCTTCTAACCCAGTAGACATCTTAACTTACGCTACATGGAAATTCTCAGGTCTTCCAAAAGAACGTGTTATCGGTTCTGGAACAAGCCTTGATACAGCACGTTTCCGCATGTCAATTGCTGACTATCTAAAAGTAGATGCTCGTAACGTCCATGGTTACATCCTTGGCGAACACGGCGATACAGAGTTCCCAGCATGGAGCCACACAACTGTCGGCGGCCTTCCAATTACTGAATGGATTAGCGAAGATGAACAAGGTGCAATGGATACTATTTTCGTAAGTGTTCGTGATGCAGCTTATGAAATTATTAATAAAAAAGGCGCTACATTCTACGGCGTTGCTGCAGCTCTTGCTCGTATTACAAAAGCAATTCTAAATAACGAAAATGCGATTTTACCACTTTCTGTTTATTTAGATGGCCATTATGGTATGAACGACATTTATAGAAAT

>SW2a_lhkA1

TGATCAGCCTTTACCTAAGGATTTCTCTATTTCTGCGGATGATAAGAAAAAGCTTGAAAGTGGTGAAACGGTTAGTAAGAAAATAGATAATCGCTTTAACAAAGAAATGACAATTGTGTACGTCCCAATAATGAATGGCGATAAATTTGTCGGTTCTATCGTGCTGAATTCACCCATTAGCGGTACGGAGCAAGTAATTGGCACGATTAACCGCTATATGTTCTACACTATTTTACTTTCTATAACGGTAGCACTTATTCTTAGCGCAATCTTGTCCAAACTACAAGTAAATCGAATCAACAAACTACGAGCAGCGACAAAAGACGTTATTCAAGGCAATTACAACGCTCGCTTGAAGGAAAATAATTTTGATGAAATTGGTGCACTCGCCATTGATTTCAATAAAATGACACAAACCCTTGAAACATCTCAAGAAGAAATTGAACGACAAGAGAAACGGAGACGCCAGTTTATTGCTGATGTTTCCCAGAAATTG

>SW941_abcZ7

TGACAGGGGTAGCTGGTAAATCGACGAACAGAATGCGTATAGGGCTTTTCCGCAAGATGGAAAAACTATCGATTCGTTTCTTCGATAGCCGCAATGATGGCGAAATGCTTAGCCGCTTCACTAGTGACTTAGATAATATTTCCAATACACTAAACCAAGCATTGATCCAAGTACTATCCAACGTCGCGCTAATGATTGGTGTTATCATCATGATGTTCCAACAAAACGTGGAACTAGCCTTCGTTACTCTAATATCTGCTCCATTTGCAATTATTATTGCGACAGTGATTATTCGAAAAGCACGTAAATTCGTTGATGTTCAACAAGATGAACTAGGCGTACTTAACGGCTACATTGACGAAAAAATCTCTGGACAAAAAATCATTATCACAAATGGTTTAGAAGAAGAAACAATTGACGGCTTTGTTAAACAAAACAATATCGTTAAAAACGCCACTTATAAAGGGCAAGTTTACTCCGGTTTACTTTTCCCAATGATGCAAGGTATTTCCCTATTAAATACAGCTATCGTTATCTTCTTCGGTGGATGGTTAGCTCTAAACGGCGACCTTGAGAAATTGT

>SW941_bglA7

CTGCTGCTGCAACCAATTCGAAGGCGCTTACAACGTCGATGGAAAAGGACTTTCCGTTCAAGATGTTACTCCAAAAGGCGGATTCGGTCACATTACTGACGGTCCAACACCAGATAACTTAAAATTAGAAGGAATCGACTTCTATCACCGTTACAAAGATGACGTGAAACTTTTTGCTGAAATGGGCTTCAAAGTTTTCCGTACTTCCATCGCTTGGTCCCGTATCTTCCCAAATGGTGACGAAACAGAGCCAAACGAAGCAGGACTAAAATTTTACGATGATTTATTCGACGAACTTCTAGCACATAATATCGAACCACTGATTACTTTATCTCATTATGAAACACCACTTCACTTATCGAAAGCTTACGACGGATGGGTAAATAGAAAAATGATCGACTTCTATGAAAACTATGTCCGCACCGTATTTAATCGGAAATTGT

>SW941_cat3

TGCTCGTGGTGCTGGTGCGCACGGGAAATTTGTTACTAAAAAAAGCATGAAAAAATATACAATGGCTAAATTTTTGCAAGAAGAAGGGACGGAAACAGAGGTTTTTGCTCGTTTTTCAACTGTAATTCATGGACAACATTCTCCAGAAACATTACGTGATCCACGAGGTTTCTCCGTTAAGTTTTATACGGAAGAGGGAAATTATGACTTTGTCGGAAATAATTTGCCAGTATTTTTCATTCGTGATGCGATTAAGTTTCCAGATGTTATTCATTCCTTGAAGCCTGACCCGCGCACAAATATTCAAGATGGCAATCGTTACTGGGATTTCTTTAGCCTTACACCGGAAGCTACGACGATGATTATGTACTTATTCAGTGATGAAGGAACGCCGGCTTCTTACCGCGAAATCCGGGGCTCTAGTGTTCATGCGTTCAAATGGATTAACGAAGAAGGCAAAACAGTTTATGTAAAATTGCGCTGGGTTCCAAAAGCAGGAATCGTCAATCTGAAAT

>SW941_dapE10

TTTGCAAAGTTGTTAGCTGAACATGGTATTGAGTCCGAAAAGGTACAATACGACGTAGACAGAGCTAGCCTAGTAAGCGAAATTGGTTCCAGTAACGAGAAGGTTTTGGCATTTTCAGGGCATATGGATGTAGTTGATGCGGGTGATGTATCTAAGTGGAAGTTCCCACCTTTTGAAGCGACAGAGCATGAAGGGAAACTATACGGACGCGGCGCAACGGATATGAAGTCAGGTCTAGCGGCGATGGTTATTGCAATGATTGAACTTCATGAAGAAAAACAAAAACTAAACGGCAAGATCAGATTATTAGCAACAGTTGGGGAAGAAATCGGTGAACTTGGAGCAGAACAACTAACACAAAAAGGTTACGCAGATGATTTAGATGGTTTAATCATCGGCGAACCGAGCGGACACAGAATCGTTTATGCGCATAAAGGTTCCATTAATTATACCGTTAAATCCACTGGTAAAAATGCCCATAGTTCGATGAAATTGTTAT

>SW941_dat5

GCGGATATCAGTTTGGTGATGGTGTATATGAAGTAGTTCGTCTATATAATGGAAAATTCTTTACTTATAATGAACACATTGATCGCTTATATGCTAGTGCAGCAAAAATTGACTTAGTTATTCCTTATTCCAAAGAAGAGCTACGTGAATTACTTGAAAAATTAGTTGCCGAAAATAATATCAATACAGGGAATGTCTATTTACAAGTGACTCGTGGTGTTCAAAACCCACGTAATCATGTAATCCCTGATGATTTCCCTCTAGAAGGCGTTTTAACAGCAGCAGCTCGTGAAGTACCTAGAAACGAGCGTCAATTCGTTGAAGGTGGAACGGCTATTACAGAAGAAGATGTGCGCTGGTTACGCTGTGATATTAAGAGCTTAAACCTTTTAGGAAATATTCTAGCAAAAAATAAAGCACATCAACAAAATGCTTTGGAAGCTATTTTACATCGCGGGGAACAAGTAACGGAATGTTCTGCTTCAAACGTTTCTATTATTAAAGATGGTGTATTATGGACGCAGAAATTGTTATCC

>SW941_ldh6

GGGGATGCAATGGATTTAAGCCATGCCGTTCCATTTTCTACACCGAAGAAAATCTACTCAGCAAATTATAGCGACTGCCACGATGCGGACCTAGTTGTTGTAACTGCCGGTACTGCTCAAAAACCTGGTGAAACTCGTTTAGATCTAGTAAATCGTAATATCAAAATCATGAAAGGCATCGTGGATGAAGTTATGGCAAGCGGATTTGATGGCATCTTCTTAATCGCTTCTAACCCAGTAGACATCTTAACTTACGCTACATGGAAATTCTCAGGTCTTCCAAAAGAACGTGTTATCGGTTCTGGAACAAGCCTTGATACAGCACGTTTCCGCATGTCAATTGCTGACTATCTAAAAGTAGATGCTCGTAACGTCCATGGTTACATCCTTGGCGAACACGGCGATACAGAGTTCCCAGCATGGAGCCACACAACTGTCGGCGGCCTTCCAATTACTGAATGGATTAGCGAAGATGAACAAGGTGCAATGGATACTATTTTCGTAAGTGTTCGTGATGCAGCTTATGAAATTATTAATAAAAAAGGCGCTACATTCTACGGCGTTGCTGCAGCTCTTGCTCGTATTACAAAAGCAATTCTAAATAACGAAAATGCGATTTTACCACTTTCTGTTTATTTAGATGGCCATTATGGTATGAACGACATTTATAGAAATTGT

>SW941_lhkA1

GATGATCAGCCTTTACCTAAGGATTTCTCTATTTCTGCGGATGATAAGAAAAAGCTTGAAAGTGGTGAAACGGTTAGTAAGAAAATAGATAATCGCTTTAACAAAGAAATGACAATTGTGTACGTCCCAATAATGAATGGCGATAAATTTGTCGGTTCTATCGTGCTGAATTCACCCATTAGCGGTACGGAGCAAGTAATTGGCACGATTAACCGCTATATGTTCTACACTATTTTACTTTCTATAACGGTAGCACTTATTCTTAGCGCAATCTTGTCCAAACTACAAGTAAATCGAATCAACAAACTACGAGCAGCGACAAAAGACGTTATTCAAGGCAATTACAACGCTCGCTTGAAGGAAAATAATTTTGATGAAATTGGTGCACTCGCCATTGATTTCAATAAAATGACACAAACCCTTGAAACATCTCAAGAAGAAATTGAACGACAAGAGAAACGGAGACGCCAGTTTATTGCTGATGTTTCCCAGAAATTG
